# Interrater agreement in atrial fibrillation diagnosis using one-week single-lead patch ECG monitoring: findings from the NORSCREEN trial

**DOI:** 10.1093/europace/euag162

**Published:** 2026-06-25

**Authors:** Marius B Haraldsen, Miroslav Boskovic, Trygve Berge, Bjørnar L Grenne, Per Torger Skretteberg, Jarle Jortveit, Sigrun Halvorsen

**Affiliations:** Department of Cardiology, Oslo University Hospital Ullevaal, Box 4950, Nydalen, Oslo 0424, Norway; Faculty of Medicine, University of Oslo, Box 1078, Blindern, Oslo 0316, Norway; Faculty of Medicine, University of Oslo, Box 1078, Blindern, Oslo 0316, Norway; Department of Cardiology, Sorlandet Hospital, Kristiansand, Norway; Department of Medical Research and Department of Medicine, Baerum Hospital, Vestre Viken Hospital Trust, Rud, Norway; Department of Cardiology and Cardiothoracic Surgery, St.Olavs Hospital, Trondheim, Norway; Department of Circulation and Medical Imaging, Norwegian University of Science and Technology, Trondheim, Norway; Department of Cardiology, Oslo University Hospital Ullevaal, Box 4950, Nydalen, Oslo 0424, Norway; Department of Cardiology, Sorlandet Hospital, Arendal, Norway; Department of Cardiology, Oslo University Hospital Ullevaal, Box 4950, Nydalen, Oslo 0424, Norway; Faculty of Medicine, University of Oslo, Box 1078, Blindern, Oslo 0316, Norway

**Keywords:** Atrial fibrillation, Screening, Single-lead patch ECG, Interrater agreement

## Introduction

Early detection of atrial fibrillation (AF) and timely anticoagulation therapy reduces stroke risk. However, anticoagulation therapy also increases the risk of significant bleeding.^1–3^ Accurate and reliable diagnosis is therefore essential to achieve an optimal benefit–risk balance in AF screening. European Society of Cardiology guidelines support the use of wearable single-lead ECG devices for AF detection, although the diagnostic reliability of brief single-lead recordings remains moderate.^1,2,4^ In contrast, single-lead patch ECG monitoring provides continuous ECG recording over several days and can capture the onset and termination of arrhythmic episodes, allowing clinicians to differentiate AF from mimics, such as frequent premature atrial contractions (PACs). Nonetheless, the reliability of AF diagnosis based on single-lead patch ECG recordings has not been firmly established.

In this study, we evaluated the interrater agreement in diagnosing AF using one-week single-lead patch ECG recordings from the NORwegian atrial fibrillation self-SCREENing (NORSCREEN) trial.

## Methods

### Study design

This predefined methodological substudy was conducted within the ongoing nationwide, randomized NORSCREEN trial, which evaluates the effectiveness of AF screening for stroke prevention in adults aged ≥65 years at increased risk of stroke.^5^

### Study device

**Figure 1:**
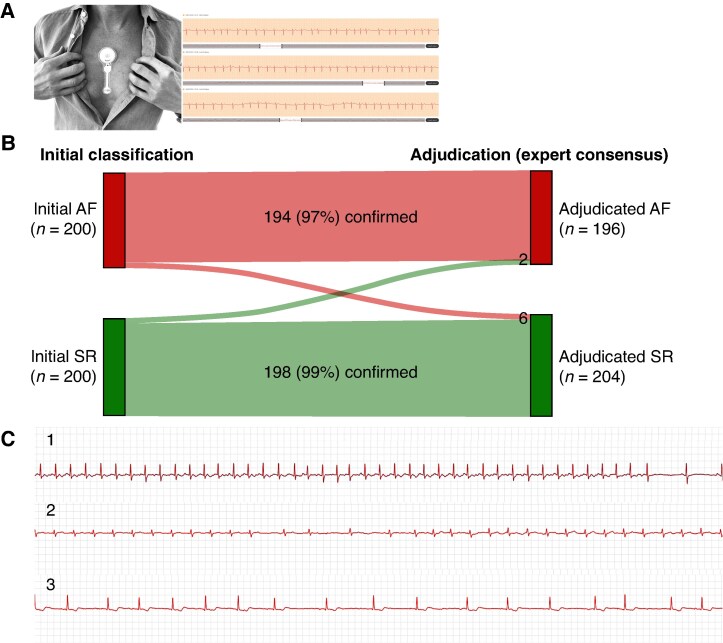
(*A*) The ECG247 smart heart sensor system. (*B*) Sankey diagram showing the results from the initial ECG interpretation and the second adjudication (expert consensus) for diagnosing or exclusion of atrial fibrillation. (*C*) Examples of ECG recordings reclassified after expert consensus: (1) atrial flutter reclassified to supraventricular tachycardia; (2) atrial fibrillation reclassified to sinus rhythm with frequent premature atrial contractions; and (3) sinus rhythm with frequent premature atrial contractions reclassified to atrial fibrillation.

NORSCREEN utilizes the ECG247 Smart Heart Sensor (Appsens AS, Lillesand, Norway), consisting of a disposable electrode patch, a reusable sensor, a medical-grade smartphone application, and a secure cloud service with real-time automated ECG analysis (*Figure 1*). The CE-marked system enables continuous ECG recording with diagnostic accuracy for AF comparable to traditional Holter ECG systems.^6^

### Study procedure

NORSCREEN is a fully digital, site-less trial with recruitment from the general Norwegian population.^5^ Inclusion criteria were age ≥65 years and at least one additional CHA_2_DS_2_-VA risk factor. Exclusion criteria were prior AF, current anticoagulation, implanted cardiac device, or lack of smartphone access. Participants were randomized (1:1) to home-based AF screening or no screening. Those randomized to screening received the ECG247 Smart Heart Sensor by mail and were instructed to wear it continuously for ≥7 days.

### Study population

Of the total NORSCREEN cohort (*n* = 50 549), this substudy included the first 200 participants with ECG recordings classified as AF, and 200 randomly selected participants with recordings initially classified as sinus rhythm (SR).

### ECG interpretation

AF was defined as an irregular rhythm without distinct *P* waves lasting ≥30 s. Atrial flutter episodes of ≥30 s were classified as AF for this analysis. We applied a two-step approach to ECG interpretation and verification:

1. The first ECG interpretation was performed combining the ECG247 automated detection and manual review: Recordings marked by the automatic algorithm as AF (≥30 s) were manually reviewed by cardiologists with access to the complete ECG recording, whereas the remaining recordings underwent screening by a trained nurse, with a cardiologist review in case of uncertainty.
2. For the second interpretation (‘expert consensus’), two experienced cardiologists independently re-evaluated all 400 complete one-week recordings. Discordant readings were adjudicated by a third cardiologist to establish a consensus AF/SR diagnosis.

### Outcomes

The outcome was the interrater agreement for diagnosing and excluding AF, measured as percent agreement between the first and second interpretations.

### Statistics

Clinical characteristics were summarized using descriptive statistics. Interrater agreement was quantified as percent agreement with 95% confidence intervals (CI). Analyses were performed using Stata 18 (StataCorp LLC, College Station, TX, USA).

### Ethics

Informed consent was obtained from all participants. The protocol was approved by the Norwegian Committee for Medical and Health Research Ethics (477781). The trial is registered at ClinicalTrials.gov (NCT05914883).

## Results

The 200 participants with ECG recordings initially classified as AF were older than those initially classified as SR (median age 75 [interquartile range (IQR) 70–78] vs. 72 [68–76] years, *P* < 0.001) and were also more likely to be men (73.5% vs. 53.0%, *P* < 0.001).

The median duration of ECG monitoring was longer in the AF group than in the SR group (172 h (IQR: 138–216) vs. 143 h (IQR: 116–180) (*P* < 0.001). Most AF cases had paroxysmal AF (*n* = 146; 73.0%), with a median AF burden of 4.0% (IQR: 1.0–10.0). During AF episodes, median ventricular rate was 110 beats/min (IQR: 90–135).

Among the 200 ECG recordings initially classified as AF, 194 were confirmed by expert adjudication, yielding 97% agreement (95% CI: 94–99%). Among the 200 recordings initially classified as SR, two were reclassified as AF, corresponding to 99% agreement for AF exclusion (95% CI: 98–100%) (*Figure 1*).

Of the six ECG recordings initially classified as AF but with subsequent disagreement, three were reclassified as supraventricular tachycardia (SVT) and three as SR with frequent PACs. For both recordings initially interpreted as SR and subsequently reclassified as AF, the first interpretation had attributed the observed irregularity to frequent PACs rather than true AF.

## Discussion

This study demonstrated a high level of interrater agreement in AF diagnosis using one-week single-lead patch ECG monitoring in a population-based screening setting, with 97% agreement for AF detection, and 99% for AF exclusion. All diagnostic discrepancies were related to differentiating AF from frequent PACs or SVT.

Reliable AF identification is essential for clinical management. In a screening setting involving predominantly asymptomatic individuals, high diagnostic specificity is critical to avoid false diagnoses and unnecessary downstream consequences. The SAFER trial has recently reported only moderate reliability using intermittent single-lead handheld devices.^4^ These results align with other studies and raise concerns about the validity of single-point, single-lead ECG for AF screening.^7–10^ The high interrater agreement in our study suggests that AF classification based on patch ECG recordings is highly reproducible and may offer advantages over intermittent ECG recording strategies. Furthermore, the high interrater agreement suggests that routine dual interpretation is unnecessary and that secondary review can be reserved for equivocal cases, improving efficiency and cost-effectiveness.

Limitations include the retrospective observational design, lack of blinding to the first interpretations, and absence of a definitive diagnostic gold standard. Further work is required to demonstrate whether the findings of this work translate to improved patient-relevant outcomes.

## Conclusions

Single-lead patch ECG monitoring demonstrated high interrater agreement for both AF diagnosis and exclusion. Its ability to differentiate AF from common mimics underscores the potential diagnostic benefits of continuous ECG monitoring and supports its use in AF screening.

## Data Availability

The data underlying this article will be shared on reasonable request to the corresponding author.
